# The Effect of Ethiopia’s Community-Based Health Insurance Scheme on Revenues and Quality of Care

**DOI:** 10.3390/ijerph17228558

**Published:** 2020-11-18

**Authors:** Zemzem Shigute, Anagaw D. Mebratie, Robert Sparrow, Getnet Alemu, Arjun S. Bedi

**Affiliations:** 1 International Institute of Social Studies, Erasmus University Rotterdam, 2518 AX Den Haag, The Netherlands; bedi@iss.nl; 2 Institute of Development and Policy Research, Addis Ababa University, Addis Ababa, Ethiopia; getnet.alemu@aau.edu.et; 3 School of Public Health, Addis Ababa University, Addis Ababa, Ethiopia; anagawd@yahoo.com; 4 Development Economics, Wageningen University, 6706 KN Wageningen, The Netherlands; sparrow@iss.nl

**Keywords:** community-based health insurance, quality of health care, revenues, Ethiopia

## Abstract

Ethiopia’s Community-Based Health Insurance (CBHI) scheme was established with the objectives of enhancing access to health care, reducing out-of-pocket expenditure (OOP), mobilizing financial resources and enhancing the quality of health care. Previous analyses have shown that the scheme has enhanced health care access and led to reductions in OOP. This paper examines the impact of the scheme on health facility revenues and quality of care. This paper relies on a difference-in-differences approach applied to both panel and cross-section data. We find that CBHI-affiliated facilities experience a 111% increase in annual outpatient visits and annual revenues increase by 47%. Increased revenues are used to ameliorate drug shortages. These increases have translated into enhanced patient satisfaction. Patient satisfaction increased by 11 percentage points. Despite the increase in patient volume, there is no discernible increase in waiting time to see medical professionals. These results and the relatively high levels of CBHI enrollment suggest that the Ethiopian CBHI has been able to successfully negotiate the main stumbling block—that is, the poor quality of care—which has plagued similar CBHI schemes in Sub-Saharan Africa.

## 1. Introduction

In June 2011, motivated by the limited increase in health care utilization, despite substantial supply-side investments in the country’s health care infrastructure [1], the Ethiopian Government introduced a pilot voluntary Community-Based Health Insurance (CBHI) scheme. The main objectives of the scheme are to increase demand for health care services, enhance financial protection and generate revenues from domestic sources for the health care sector [2]. Several reviews of the CBHI literature [3,4,5,6] show that while such voluntary insurance schemes have had some success in enhancing financial protection and enabling access to health care, they struggle to expand enrollment and to retain clients.

Based on a systematic review of 46 CBHI studies conducted in low- and middle-income countries, Ref. [7] reports an unweighted average insurance uptake rate of 37% and high dropout rates. For instance, for a scheme in Guinea-Conakry, Ref. [8] report a dropout rate of 25%. In the case of the Nouna district scheme in Burkina Faso, depending on the year, the dropout rate ranged between 31 and 46% [9] while in Senegal, scheme dropout rates ranged between 58 and 83% for three schemes set up between 1997 and 2001. While there are several factors that lead to scheme dropout, in almost all the papers, the (perceived) quality of care on offer has appeared as a prominent reason inhibiting continued enrollment. For example, in their work on Guinea-Conakry, Ref. [8] concluded that while affordability was an issue, the main reason for the declining enrollment rate was the poor quality of care. Similarly, in their assessment of a scheme in Burkina Faso, Ref. [9] identified quality of care as perceived by household heads as an important aspect determining dropout and, in Senegal, Ref. [10] reported that a negative perception of quality of care increased the probability of dropping out. Despite the importance attributed to quality of care in influencing enrollment and dropout, the literature on the effects of CBHI schemes on generating revenues and on quality of care is scarce [6].

This paper is motivated by the relatively limited literature on the effects of CBHI on revenue generation and quality of care, and the potentially strong link between quality of care and CBHI uptake and retention, which has implications for the sustainability of CBHI schemes. The main objectives of the paper are to examine the effect of being a CBHI-affiliated health center on the volume of patients accessing health care services, followed by an assessment of the scheme on resource mobilization and on two types of quality measures—perception-based measures and indicators of structural quality. Finally, we analyze the effect of CBHI-affiliated health centers on patient satisfaction. At the outset, it is important to clarify that while this paper relies on arguably relevant measures of quality, we are well aware that there is a difference between, for instance, the availability of medical equipment in a health center (which is what we measure) and correct use of the equipment or similarly information on the number of staff supposed to be at a health center (what me measure) and staff actually present. We also do not have a good sense of the knowledge of health workers and what they actually do in practice (for instance, see [11,12]. Keeping in mind this caveat, the paper is based on two rounds of health facility survey data collected in 2011 and 2014 from 48 health centers and three rounds of household survey data collected in 2011, 2012 and 2013. The analysis focuses on health centers, that is, the middle-rung of the health care system since these are the first port of call for accessing curative care. Such centers also regulate entry to higher levels of the health care system. Health posts provide care for free and services from privately run clinics are not covered by the scheme.

The next section of this paper contains a conceptual discussion of quality of care, a review of the relevant literature and a brief review of the Ethiopian CBHI scheme. Section 3 discusses data and methods, Section 4 presents results and the final section contains a discussion and concluding remarks.

## 2. Materials and Methods

### 2.1. Conceptualizing Quality of Care

While the main objectives of the paper are straightforward, the concept of quality of care and the indicators that may be used to measure quality are more intricate. Consolidating different definitions, the World Health Organization (WHO) defines quality of health care as “the extent to which health care services provided to individuals and patient populations improve desired health outcomes” [13]. This definition calls for a focus on health outcomes which is certainly the end goal of a health care system but it is perhaps not so useful with regard to measuring the proximate effects of insurance-related interventions (such as health care utilization, quality of health care) which in turn may translate into better health outcomes.

In one of the earliest papers to provide a more comprehensive conceptualization of quality of care and suggest ways of measuring it, Refs. [14,15,16] proposed a systems-based framework of structure, process and outcome to capture the various dimensions of health care quality. Structure refers to the availability of physical facilities, equipment, drugs and human resources. Process refers to the manner in which these facilities and human resources are deployed in clinical terms (for instance, whether medical procedures are followed, appropriate tests are conducted), as well as interpersonal handling of patients which includes the gamut of interactions between client and practitioner during the care process. The final dimension refers to the translation of structure and process into outcomes such as morbidity, mortality, restoration and function as well as, at least in current debates, patient well-being and satisfaction. Various versions of Donabedian’s approach have been used as a basis for defining quality [17,18].

Rather than focusing on the system, Ref. [17] argue in favor of a focus on the individual user of health care services and suggest two dimensions of quality. These include access to services (do users receive the care they need?) and effectiveness (is the care effective when they receive it?). Effectiveness is further divided into clinical effectiveness and the effectiveness of interpersonal care. While emphasizing the placement of patients at the center of quality considerations, Ref. [17] persist with the use of the three domains—structure, process and outcome.

In a departure from a focus on structure, process and outcomes, [19], along with a series of other authors [20,21,22,23,24], argue in favor of a predominantly patient-centered approach to measuring quality of care. The argument is that from a patient’s perspective, patient satisfaction is the ultimate end point of what a health care system is trying to achieve and a direct assessment, albeit subjective, of such satisfaction should be an essential part of quality assessment.

Drawing on the conceptual literature on quality of care and based on the available data, the current study examines the effect of the CBHI on structural measures of quality (e.g., availability of drugs, equipment), process (essentially waiting time) and patient satisfaction.

### 2.2. The Effects of CBHI on Revenue Generation and Quality of Care

While there is a large body of literature that has examined the effect of CBHI schemes on utilization and financial protection, the literature on the effects of such schemes on revenues and especially on quality of care is relatively scarce. In a review of 36 studies, Ref. [4] identifies seven studies that examine the effect of CBHI schemes on cost-recovery ratios, usually defined as the share of recurrent expenditures incurred by a provider that are met through insurance pay-outs, and one study that examines effects on quality of care. The study concludes that such schemes have moderate effects on cost-recovery (cost-recovery ratio of 25%) and no evidence that they have an effect on quality of care. In a similar study, Ref. [5] find a modest effect of CBHI schemes on revenue collection, resource pooling and purchase of care services.

A recent and more comprehensive review which is not restricted to CBHI schemes is provided by [6] (which assesses the effects of community-based health insurance, social health insurance and private health insurance schemes). In their review of 159 studies that analyzed the effect of insurance schemes in Asia (91 schemes) and in Africa (68 schemes), the authors identified 19 studies that deal with the effects of CBHI schemes on resource mobilization and 8 studies that deal with CBHI effects on quality of care. They find that in 13 of the 19 studies there is a strong positive effect on resource mobilization. Methodologically, the papers compare revenues raised through CBHI versus revenues raised through user fees [25,26,27] or provide an assessment of changes in revenues raised or changes in cost-recovery ratios over time [28,29,30,31]. Based on the evidence, they conclude that the evidence is credible enough to recommend CBHI schemes as an alternative to user fees in health care financing and a promising means to achieve universal health care coverage.

In contrast to the strong effects on resource mobilization, Ref. [6] find that CBHI schemes have weak positive effects (4 out of 8 studies), if any effect at all, on structural and perceived quality of care indicators. For CBHI schemes in Africa the structural quality of care outcome indicators included drug availability, number of technical staff, staff time per visit, range of services offered, waiting time and consultation by nurse instead of doctor and patient perceptions of drug availability, the quality of the prescribed medicine and staff attitude. The papers are based on either cross-section data or only on qualitative information. Three of the papers which find positive effects are on Rwanda’s CBHI while the additional paper focuses on Tanzania. These papers show that pre-payment schemes are associated with greater drug availability in both Rwanda and in Tanzania.

This paper adds to the literature on the effect of CBHI schemes on revenue generation and on quality of care. The paper relies on panel data at the health facility level, as opposed to cross-section data, and on three rounds of household survey data. In addition to the use of panel data, the paper contributes by examining the effect of such schemes on both structural indicators of quality and on patient satisfaction. Moreover, in contrast to earlier studies, we examined the effect of the CBHI on quality from the perspective of both providers and patients.

### 2.3. Health Care Financing in Ethiopia and the CBHI: A Brief Overview

In 1998, the Ethiopian government developed a health care financing strategy (HCFS). This strategy had four interrelated objectives—identification of additional resources for the health sector, mobilization and deployment of these resources to the health sector, enhancing resource efficiency and ploughing additional resources to enhance the quality of care. Emanating from the HCFS, a number of reforms have been implemented including increases in user fees, fee retention and utilization of generated resources by care providers, and the introduction of health insurance schemes.

In 1995/96, health care spending amounted to 3.5% of GDP with per capita health spending of USD 4.5. The bulk of the resources came from households (53%), while federal and regional governments contributed 40% [32]. In contrast, after the implementation of the HCFS, in 2013/14, health care spending amounted to 4.7% of GDP and per capita health spending had risen to USD 28.7. At the same time the share of out-of-pocket (OOP) expenditure fell from 53 to 33%, with the share of the government constituting about 30% and the remainder coming from international sources (Table 1). In terms of the contribution of the CBHI, the 2013/2014 NHA document states that, “the current HA estimation looks at 2013/14, too soon for the Ethiopian Health Insurance Agency to show a significant contribution in managing health resources. At that time, the community-based health insurance (CBHI) had been piloted in only 13 woredas, in the four largest regions, and preparation of the social health insurance (SHI) program was just starting.” [33].

In its continuing attempts to enhance the sustainability of health care financing and to increase reliance on domestic sources, the government introduced a voluntary CBHI for rural areas and informal sector workers in urban areas. The CBHI was introduced on a pilot basis in June 2011 in 13 districts located in four main regions of the country (Tigray, Amhara, Oromia, and SNNPR). It was offered to about 301,000 households at a premium of Ethiopian Birr (ETB) 126 to 180 per year for core household members (core household members include parents and their children below the age of 18). The premiums amount to 0.5 to 1% of household monthly income. Households pay premiums and a one-time registration fee to a village office which then transfers the funds to the CBHI scheme. The Woreda CBHI scheme carries out four main responsibilities: (i) identifying and signing contracts with health facilities to provide care to scheme beneficiaries, (ii) reimbursing providers for the health care utilized by beneficiaries, (iii) financial administration and (iv) database management including membership, premium payment and utilization of health care by members. Scheme enrollees may access both outpatient and inpatient health care services in public facilities which have signed a contract with the CBHI scheme. In preparation for service provision, resources were provided in kind and in cash to contracted health facilities by the federal government. This support amounted to ETB 40,000 and was meant to ensure an acceptable level of quality of health care, that is, support the purchase of drugs, medical supplies and/or medical equipment. Woreda and regional government also contributed to enhancing the capacities of contracted facilities in terms of human resource and basic infrastructure, including water, electricity/generator [34]. There are no co-payments and use of services is free at point-of-use (additional details on the scheme are available in [35]).

As of June 2014, according to administrative data [1] more than 50% of households in the pilot districts had joined the scheme (157,553 of 300,799). Analysis of household survey data shows a similar picture with enrollment rising from 41% in 2012 to 48 in 2013 and to 58% in 2015 (Table 2). While households do dropout (18% in 2013 and 19% in 2015), this is more than compensated by the induction of new enrollees (25% in 2013 and 28% in 2015). Across regions in 2015, enrollment ranged from 48% in Tigray to 68% in Amhara. Consistent with this pattern, drop-out rates are also higher in Tigray as compared to the other regions. Despite this variation, what is notable is that across all regions enrollment rates are relatively high as compared to those noted in the literature [7].

Administrative data also show that between the scheme launch and June 2014, the CBHI scheme had received about ETB 30 million as membership contributions. Additionally, according to [34], utilization of health care rose by at least 30% and the frequency of visits by at least 45%. Whether the high rate of enrollment, the pre-payment contributions and increases in utilization translate into greater revenues for CBHI-affiliated health centers and subsequently enhance the quality of care is explored in the succeeding sections.

### 2.4. Health Facility Surveys

The first health facility survey, a baseline round was conducted in 2011 and a second follow-up survey, after the introduction of the CBHI, was conducted in 2014. In both years, the surveys gathered information on 48 health centers, 3 in each of the 16 districts. Of these 16 districts, 12 are districts where the CBHI was offered and four are districts where it was not offered. On average, in 2010–2011, there were about 3.3 health centers per district (nationwide) [36]. This number was calculated by dividing the total number of health centers (2660) by the total number of districts (800) in the country in 2010–2011 [36]. Thus, our surveys cover almost all the health centers in these 16 districts.

The health facility surveys gathered data from the head of the health center as well as from administrative records. The surveys gathered monthly data on outpatient and inpatient visits for a period of 12 months preceding the survey, annual revenues accruing from government transfers, patient cards, diagnostic services, sales of drugs and other sources, also for the 12 months preceding the survey. With regard to structural measures of quality, we obtained (i) information on the number of staff and their education levels (we also have information on the location, year of establishment, individual characteristics of the head of the health center (age, sex, education, training and experience); these variables are used as controls in the statistical work), (ii) information on the availability of 18 essential drugs, (iii) availability of 21 types of equipment and facility including laboratory services (blood, urine, stool and rapid HIV tests), an outpatient care team, delivery care, maternal and child health service, emergency care and an inpatient medical service team and (iv) access to water and electricity. In addition to these structural availability indicators, respondents were asked to identify and list the main weaknesses in their ability to deliver services. These perception-based indicators included options such as shortage of drugs, lack of medical equipment, shortage of financial resources and inadequate water supply. Finally, exit interviews of five patients per facility provided information on the time taken to obtain a patient card and the time taken to see a health professional (nurse, doctor or health officer). The responses on waiting time were used as process indicator outcomes. The first part of our analysis, reported in Section 3.2 and Section 3.3, relies on data from these health facility surveys (Appendix A, Table A1 provides a description of the health facility variables). In principle, since 48 health centers were surveyed twice, we should have 96 observations for each of the outcomes. However, for a number of the outcomes we were not able to obtain data from all 48 health centers. This is especially with regard to data on revenues where observations range between 16 and 74. For the various quality measures the data are relatively complete.

### 2.5. Household Survey Data

Three rounds of household data were collected, with the first round in March–April 2011, that is, a few months before the CBHI launch, and the subsequent rounds were collected in March–April 2012 and March–April 2013. The surveys were fielded in the same sixteen districts in which the health facility surveys were conducted. Within each district, six villages were randomly selected and within each village 17 households were randomly surveyed, yielding a sample of 1632 households. The surveys contained various modules covering individual and household socioeconomic characteristics, demographic traits, health status and health care utilization. Of particular interest for this paper, the survey included a question on patient satisfaction with the health care received on a Likert scale ranging from unsatisfied to satisfied (for respondents that used health care). We focus on patient satisfaction regarding the use of outpatient care from health centers in the two months preceding the survey. This yields a total of 1156 individual observations spread over three years. The numbers differ across each of the three years and we have 410 observations for 2011, 345 for 2012 and 401 for 2013. Each of these observations was linked to the health center closest to their place of residence. Some of the health centers had signed contracts with the CBHI scheme in 2012 while others had not (Appendix A, Table A2 provides a description of the household variables).

### 2.6. Empirical Framework

#### 2.6.1. Health Facility-Based Outcomes

Our primary aim is to detect the effect of the CBHI on revenues and quality of care. Prior to examining the effect on these outcomes we examined the effect of being affiliated with the CBHI on use of health care as captured by the monthly volume of patients (averaged over twelve months) visiting health care facilities for outpatient care, followed by the effect of CBHI affiliation on various revenue sources (the revenue data are in real 2011 prices). This is followed by an examination of the effect of CBHI affiliation on a range of health care quality indicators. These include structural quality measures, (availability of drugs, medical equipment/facilities and access to water and electricity), perception-based quality indicators (drug shortages, budget shortfalls, self-assessed overall quality of service) and finally waiting time to obtain a patient card and to meet a medical professional.

We assessed the effect of being affiliated to the CBHI on these outcomes using a difference-in-differences approach. That is, we exploited the longitudinal nature of the data and estimated a health facility model which contains both fixed and temporal components,

(1) $$y_{jt}=\alpha +\beta CBHI_{j}*d2014_{t}+X_{jt}\delta +\pi d2014_{t}+\theta_{j}+\varepsilon_{jt}$$

where $y_{jt}$ indicates the outcomes of interest for facility *j* at time *t*, $X_{jt}$ is a matrix of time-varying controls, *d2014* indicates the time period of the observation (2011 or 2014) and the interaction term, $CBHI_{j}*d2014_{t},$ indicates whether facility *j* is affiliated with the CBHI after it was introduced. In the absence of further control variables, the parameters of the model capture the difference-in-differences estimates: β reflects the impact of the CBHI scheme, α the baseline outcome for the control group, while π is the time trend for the control group. The facility group indicator $CBHI_{j}$ is absorbed by the health facility fixed effects $\theta_{j}$ that control for time-invariant observable and unobservable health facility traits, including any initial differences between the CBHI and non-CBHI facilities, that could bias the estimates. Provided that the time-variant error term $\varepsilon_{jt}$ is uncorrelated with the other covariates in Equation (1), this specification will yield unbiased estimates of the effect of the CBHI scheme. This identifying assumption implies that, in the absence of the CBHI scheme, the treatment and comparison group would have experienced similar (or parallel) trends in the outcome variables. This assumption would seem appropriate since the decision to roll out the CBHI scheme was not based on trends or shocks, and the main source of bias is expected to come from initial differences between facilities. In addition, the panel data regressions allow us to control for time-variant observable traits. The results are robust to adding controls, so in the remainder of the paper we present the results without control variables (the results displaying the coefficients on the fixed effects and with control variables are available upon request). A limitation of our data set is that the relatively small number of observations affects the power of our estimates to detect causal effects.

#### 2.6.2. Household Survey Outcomes

With regard to the household survey data, we treat patient satisfaction (satisfied or not satisfied) as a function of a matrix ($Z_{ijt}$) of observed time-varying covariates (socioeconomic characteristics, demographic factors, health status, travel time to the nearest health center, financial participation and networks and location dummies), time fixed effects, and whether the health center closest to the residential location of patients has a CBHI affiliation ($CBHI_{j}$) (it is possible but improbable given the transportation and road infrastructure in rural Ethiopia, that households seek care at centers other than their most proximate health center). Since we have data from a period of over three years, we estimated a model with 2011 as the base year and 2012 and 2013 as the follow-up years. Thus, we estimated the satisfaction of patient *i* with health services offered in health facility *j* at time *t*, that is, ($PS_{ijt}$), as follows:

(2) $$\begin{array}{c} PS_{ijt}=\alpha +\beta_{2012}CBHI_{j}*d2012_{t}+\beta_{2013}CBHI_{j}*d2013_{t}+Z_{ijt}\delta \\ +\gamma_{2012}d2012+\gamma_{2013}d2013_{t}+\rho CBHI_{j}+\varepsilon_{ijt} \end{array}$$

The coefficients on the two interactions terms are the main parameters of interest and capture the change in satisfaction over time for those individuals who receive treatment from CBHI-affiliated facilities. The variables $d2012_{t}$ and $d2013_{t}$ are dummy variables for each of the two time periods. In this household analysis, the $CBHI_{j}$ facility variable now captures the initial differences between the CBHI and control facilities (reflected in parameter ρ). We estimated variants of Equation (2) using a linear probability model.

All statistical analyses were performed using Stata 15. Both the data and the code used to generate the results are available on request.

## 3. Results

### 3.1. Descriptive Statistics

Of the 48 health centers, 36 have signed contracts with the CBHI scheme. Descriptive statistics of the variables of interest in 2011 and 2014 are provided in Table 3. Prior to the introduction of the CBHI, contracted health centers recorded 590 patient visits per month per health center in the 12 months preceding the survey while for non-contracted centers the corresponding number was a little higher at 637 visits per month. In 2014, patient visits to contracted centers almost doubled to 1073 visits per month while for non-contracted centers there was almost no change (616 visits per month). Consistent with this increase, there was a sharp jump in revenues from patient cards, diagnoses, drug sales and total revenues at contracted health centers. With regard to government revenues, there is a decline for health centers affiliated to the CBHI but this is more than compensated through revenues accruing from insurance payments. The revenues are also the only aspect in which the CBHI and the non-CBHI group show initial differences, as CBHI facilities have higher revenues from government budget and drug sales in 2011. This suggests that the fixed effects approach is justified, since cross-section differences would overestimate the impact of CBHI.

With regard to structural indicators of quality, in 2011, in both contracted and non-contracted health centers, 15 of 18 types of drugs were available. In 2014, there was a slight increase and, in both groups, 16 of 18 drug types were available. A similar picture emerges for medical equipment availability. Across both years and both groups, health centers have 15 to 16 of the 21 types of equipment that they are expected to have. Despite the similarity in the availability of drugs across contracted and non-contracted facilities, the perception of health care workers with regard to the availability of drugs is markedly different across the two groups. In 2011, in 19% of the contracted health centers, workers perceived drug shortages while this dropped to zero in 2014. Similarly, with regard to budget shortfalls, health care worker perceptions of shortfalls dropped from 17 to 3% in contracted facilities while it rose from zero to 8% in non-contracted facilities (although this is not reported in the table, there seems to have been a general increase in staff for both groups of health centers over time. The total number of medical staff in health centers increased from 25 to 31 workers on average, while the number of technical staff increased from 17 to 20).

### 3.2. Effect of CBHI Affiliation on Patient Volume and Revenues

Estimates of the effect of CBHI affiliation on outpatient visits and revenues are in Table 4. While there is no change in the outcomes over time for the control group (coefficient on the time dummy is insignificant), consistent with the descriptive statistics, the estimates show that, over time, after controlling for health center fixed effects, CBHI-affiliated health facilities experience a sharp increase in the volume of outpatient visits (coefficient on the interaction term). The increase amounts to 653 more outpatient visits per month or an increase of 111%, as compared to outpatient visits to contracted health centers in 2011. As may be expected, given the sharp increase in patient visits, there is an increase in resource flows to contracted health centers mainly from patient cards and drug sales. As compared to the baseline for the contracted group, signing a contract is associated with a 178% increase in revenues from patient cards and a 75% increase in revenues from drug sales. Although health facilities were not always able to provide revenue breakdown by types of revenue, a larger number of the health centers were able to provide information on total revenues including revenue flow from the CBHI scheme. As shown in the last column of Table 4, total revenues increased by ETB 239,000 (in real terms) or an increase of 47% as compared to the baseline figure for contracted facilities. Although not shown in the table, the estimates are robust in terms of the inclusion of covariates such as the number of staff and characteristics of the head of the health facility.

### 3.3. Effect of CBHI Affiliation on Quality of Care

Where are the additional resources spent? Table 5 provides information on the manner in which CBHI-generated resources are spent. The most common use is to purchase drugs and disposable medical equipment such as syringes, gloves and other related items. About 90% of the facilities report using the resources for these two purposes. This is followed by efforts made to purchase durable medical equipment, cover utility costs and enhance access to water and electricity. Although, the retained fees are not meant to be used for salaries and payments, about one-fifth of the facilities do use the additional resources to reward staff.

Despite reporting that the additional resources were used to buy drugs and (durable) medical equipment, there is no association between CBHI affiliation and drug and equipment availability, at least as measured by the binary question of whether a particular set of drugs or a particular equipment is available or not (Table 6) (although not presented here, in the case of both Table 6 and Table 7, we also estimated specifications controlling for the characteristics of the head of the health center and the number of medical staff. Results were not substantially different. These are available on request). This is perhaps not surprising as the availability of drugs and equipment in both contracted and non-contracted health centers was already high in 2011 and did show much change in 2014. Furthermore, such binary outcome variables do not capture whether drugs are always available or the volume of availability. Indeed, in contrast to the responses to these objective variables, the perception of whether there is still a problem of drug shortages declines by 28 percentage points for CBHI-affiliated facilities. Similarly, the perception that there is a general shortage of financial resources declines by about 22 percentage points (Table 7).

Despite the large increase in outpatient visits at CBHI-contracted facilities there is no statistically discernible effect in waiting times to obtain patient care or see a medical professional. This may seem surprising but despite the increase in the number of outpatient visits, utilization of health care remains low in Ethiopia [35] and at the same time there has been an increase in the number of medical and non-medical staff across all health centers.

### 3.4. Effect of CBHI on Patient Satisfaction

Descriptive statistics of patient satisfaction for each year are in Table 8 while ordinary least squares (OLS) estimates of the impact of CBHI affiliation on a dichotomous (satisfied or not) specification of patient satisfaction are in Table 9 (Table A3 provides estimates based on a comparison between 2011 and 2013, excluding 2012. Differences between the results presented in Table 9 and Table A3 are minor). Column 1 of Table 9 provides estimates without the inclusion of individual controls while the remaining columns control for a range of individual attributes. At baseline, CBHI-contracted health centers appear to offer lower patient satisfaction and the time dummies indicate that non-contracted health facilities tend to offer lower patient satisfaction in both 2012 and 2013 as compared to 2011. In contract, regardless of the specification, the interaction terms indicate that there is a positive association between CBHI affiliation and patient satisfaction in 2013. Between 2011 and 2012, there was no change—in fact, the descriptive statistics show a dip in the case of both contracted and non-contracted facilities. However, by 2013, CBHI affiliation of health centers is associated with an increase in patient satisfaction by 11 percentage points. Controlling for individual traits increases this to 18 percentage points. As evident from the descriptive statistics, the increase is driven by a relative decline in satisfaction with services offered at non-contracted facilities between 2011 and 2013. While it is hard to pinpoint the reasons for the relative increase in satisfaction, the increase in resource flows to the CBHI-affiliated health centers combined with the spending patterns of the centers and the perception that drug shortages and budget shortages have declined, suggests that the increase in patient satisfaction is driven by the higher quality of care on offer.

## 4. Discussion

This paper examined the effect of health facility affiliation to a CBHI scheme in Ethiopia on the volume of outpatient visits, resource mobilization and quality of health care. The paper was based on a two-round panel of 48 health facilities and three rounds of household data. Consistent with [35], we find a sharp increase (111%) in the number of outpatient visits to CBHI-affiliated health centers. The increase in patient flows was accompanied by increases in health center revenues of 47%. As part of a virtuous cycle, the increased revenue flow was predominantly used by health centers to purchase drugs and disposable and durable medical equipment. We also found a positive effect on patient satisfaction. Patients treated at CBHI-affiliated health centers were 11 percentage points more satisfied than those treated elsewhere.

While the results obtained here are consistent with the effects of such schemes in generating revenues, they are different when it comes to effects on quality of care. A recent review of the literature [6] finds that in 13 of 19 studies there is a strong positive effect of such schemes on resource mobilization. The results here back such a claim. Perhaps more importantly, from the point of view of sustaining such schemes, we find that unlike the literature [6] which shows that such schemes have weak positive effects on structural and perceived quality of care indicators, the Ethiopian scheme stands out and is associated with an increase in patient satisfaction.

The bulk of the existing literature on CBHI schemes in Sub-Saharan Africa has argued that high dropout rates are driven by the low quality of care. For instance, Refs. [8,9,10] concluded that the main reason for the declining enrollment rate was the poor quality of care. The Ethiopian CBHI scheme offers a marked contrast. The results presented in this paper combined with the relatively high levels of CBHI enrollment, retention and re-enrollment of those who had dropped out (Table 1) suggests that the Ethiopian CBHI has been able to successfully negotiate the main stumbling block, that is, the poor quality of care, which has plagued similar CBHI schemes in Sub-Saharan Africa. The combination of supply-side investments prior to launching the CBHI and the freedom to use retained resources to finance health facility expenditure is likely to have been contributory factors.

A pertinent question here is, how much does the scheme contribute to revenue generation? Based on the current premiums, the contribution of the scheme to the overall health care budget is likely to remain limited. For instance, as of June 2019, according to the Ethiopian Health Insurance Agency, the scheme provided services in 507 districts and had an enrollment of 4.9 million households. Even if the scheme was to be expanded to the entire country and had an enrollment rate of 50% of all households (about 8 million households), based on the current premiums the scheme would be able to generate resources to cover about 5% of the government’s on-budget health expenditure of ETB 24.5 billion for 2015/16 [37]. While this may seem small, it is a start.

While these results are promising, they are based on a relatively small sample and on data that were collected in 2011 and 2014. This is a limitation of the current study and an update on the basis of larger and more recent data is needed. To emphasize, the data we do have covers almost all the health facilities in the districts that were included in the CBHI household survey, and offer sufficient variation across facilities and over time to identify the effects of the CBHI scheme. However, the relatively small number of observations reduces the statistical power of our estimates. Nevertheless, the paper contributes to the literature by examining the effects of such schemes on outcomes that have not been examined as often—most importantly, quality of care, which has been identified as the key reason for the lack of success of such schemes. Thus, the current analysis suggests that such schemes are able to enhance quality of care and generate resources for the health sector.

## 5. Conclusions

This paper used a difference-in-differences approach applied to two rounds of health facility survey data and three rounds of household survey data to examine the effect of a CBHI scheme introduced in Ethiopia on health care utilization, health facility revenues and different dimensions of quality of care. The main conclusions are that the scheme is associated with an increase in health care utilization, an increase in revenue generation and an increase in patient satisfaction. Despite the small sample size and limited statistical power, these results and the relatively high levels of CBHI enrollment do support the idea that the Ethiopian CBHI has been able to successfully deal with the main stumbling block, that is, the poor quality of care, which has plagued similar voluntary health insurance schemes in Sub-Saharan Africa. The results of this paper along with the existing work on the Ethiopian CBHI suggest that, as in the case of Rwanda and Ghana, the Ethiopian CBHI may play an important role in enhancing access to health care to wide swathes of the country’s population.

## Figures and Tables

**Table 1:** Health care financing in Ethiopia.

	1995/96	1999/2000	2004/05	2007/08	2010/11	2013/14
Share of health care spending to GDP (%)	3.5	4.4	5.2	4.5	5.2	4.7
Per capita health expenditure (USD)	4.5	5.6	7.1	16.1	20.8	28.7
Source of financing (%)						
Government including parastatals	40	33	31	22	16	30
Households (mainly OOP)	53	36	31	37	34	33
Rest of the world	1	22	37	39	50	36
Others	7	9	2	1	1	1

Source: National Health Accounts.

**Table 2:** Enrollment and drop-out across the pilot regions—community-based health insurance (CBHI) Ethiopia (%).

		Region				Total
		Tigray	Amhara	Oromia	SNNPR	
2012						
Enrollment	%	33.9	49.5	44.2	35.3	40.7
	N	101	148	133	107	489
2013						
Enrollment	%	50.2	62.7	44.5	35.4	48.2
	N	146	188	133	107	574
Drop-outs	%	26.5	6.9	21.2	21.5	18
	N	26	10	28	23	87
New enrollees	%	38.3	33.8	17.4	11.8	25.1
	N	74	52	29	23	178
2015						
Enrollment	%	48.1	68.4	55.6	58.6	57.7
	N	136	201	164	116	617
Drop-outs (2013 members)	%	31.5	9	18	17.8	19.1
	N	46	17	24	19	106
New enrollees (2012 and 2013 non-members)	%	21.9	30.4	31.2	30.1	28.3
	N	25	31	43	22	121
Re-enrollment (2013 dropouts)	%	53.9	54.6	57.1	47.8	53.4
	N	14	6	16	11	47

Source: Household surveys 2012 to 2015. Notes: In 2012, there were 489 insured households. With 87 drop-outs and 178 new entrants the total enrolled in 2013 should be 580. However, we report 574 because, of the 489 households enrolled in 2012, one did not report its enrollment status in 2013 and five were not resurveyed in 2013. Of the 574 households insured in 2013, insurance status in 2015 was not reported by 6 households and 13 households were not resurveyed. Thus, instead of 636 (574 + 121 + 47 − 106 = 636) enrolled households in 2015, we observed 617 households.

**Table 3:** Descriptive statistics: comparisons based on health center contract signing status.

	2011					2014				
	Contracted		Non-Contracted			Contracted		Non-Contracted		
Variables	Mean	SD	Mean	SD	*p*-Value	Mean	SD	Mean	SD	*p*-Value
**Monthly volume of outpatients**	590	652	637	576	0.88	1073	760	616	495	0.12
**Revenue (in ‘000 ETB)**										
Patient card revenue	24.3	40.2	31	36.7	0.73	48.2	48.1	15.5	9.4	0.03
Diagnosis revenue	4.3	6.8	18	18.2	0.03	21.3	27.1	23.6	39.7	0.86
Drug revenue	143.3	172.4	62.6	45.6	0.18	220.1	157.9	77.5	37.9	0.005
Government budget revenue	348.1	294.2	68.8	45.7	0.07	218.1	341.2	37.2	25.3	0.15
CBHI revenue	.	.	.	.	.	202.7	235	.	.	.
Total revenue-reported (including money from CBHI)	500.2	463.3	126.3	67.7	0.02	716.4	609.8	103.5	47.9	0.00
**Availability**										
Availability of drugs (18 in total)	14.8	1.80	15.3	2.5	0.51	15.8	1.6	15.8	1.7	0.96
Availability of equipment (21 in total)	15.8	2.4	16.7	1.6	0.25	16.7	1.8	15.7	1.6	0.23
**Infrastructure**										
Water supply	0.53	.	0.58	.	0.74	0.81	.	0.83	.	0.84
Electricity access	0.81	.	0.75	.	0.69	0.94	.	0.83	.	0.24
**Self-reported problems**										
Shortage/poor supply of drugs	0.19	.	0.00	.	0.10	0.00	.	0.08	.	0.08
Shortage of budget	0.17	.	0.00	.	0.14	0.03	.	0.08	.	0.42
**Average waiting time (in min)**										
To get medical card	11.7	10.6	15.8	7.9	0.25	12.4	11.1	27.3	30.2	0.01
To see medical doctor	32.1	27.9	31.3	11.7	0.92	21.5	23.2	19.7	17.3	0.81

Notes: Figures are based on the health facility surveys. The number of observations differs across variables. In 2011, the number of observations ranged between 17 and 48 while in 2014 the number of observations ranged from 26 to 48 observations. *p*-values are for mean difference comparisons between contracted and non-contracted health centers. Revenue indicators in 2014 are adjusted for inflation.

**Table 4:** Effects of CBHI affiliation on outpatient volume and revenues.

Variables	Monthly Volume of Outpatients	Revenue from Patient Card (‘000 ETB)	Revenue from Diagnosis (‘000 ETB)	Revenue from Drug Sales (‘000 ETB)	Revenue from Government Budget (‘000 ETB)	Total Revenue Reported + CBHI (‘000 ETB)
CBHI*d2014	653 ***	43.3 **	−16.1	107.4 ***	−125.5	239.1 **
	(192)	(16.6)	(27.7)	(28.6)	(81.0)	(92.9)
Time period—2014	−95	−12.8	21.2	0.50	−21.9	−22.9
	(146)	(12.1)	(26.7)	(14.1)	(15.0)	(19.7)
Constant	595 ***	29.8 ***	15.4 *	121.8 ***	329.0 ***	409.3 ***
	(52)	(4.2)	(6.9)	(9.6)	(35.0)	(34.4)
Observations	50	32	16	66	50	74
Adj-Rsq *p*-value (inclusion of health center fixed effects)	0.479 0.000 ***	0.351 0.001 ***	0.042 0.275	0.417 0.000 ***	0.109 0.028 **	0.153 0.000 *

Notes: The analysis is restricted to health centers that provided information on the particular outcome variables in both 2011 and 2014. In a number of cases, health centers were able to provide information on total revenues but not on each revenue item. Robust clustered standard errors in parentheses. All specifications control for health center fixed effects. Statistical significance: *** *p* < 0.01, ** *p* < 0.05, * *p* < 0.1.

**Table 5:** Utilization of CBHI-generated resources in 2014.

	(%)	N
Drugs	94.4	34
Disposable medical facilities (syringes, bandages, medical gloves, detergent)	88.9	32
Durable medical equipment	77.8	28
Utility payments (electricity, telephone, water)	52.8	19
Improve infrastructure (water, electricity)	47.2	17
Upgrade or expand construction	41.7	15
Salaries and incentives to employees	22.2	8
Transferred to government finance office	11.1	4

Notes: The figures show the proportion and number of health centers that spend CBHI-generated resources on a particular line of expenditure. There are 36 health centers affiliated to the CBHI scheme.

**Table 6:** Effects of signing CBHI contract on availability of drugs, medical equipment/facilities and basic infrastructure.

Variables	Drug Availability	Medical Equipment/Facility Availability	Water Supply	Electricity Access
CBHI*d2014	0.023	0.035	0.028	0.056
	(0.060)	(0.032)	(0.154)	(0.157)
Time period—2014	0.032	0.020	0.250 *	0.083
	(0.057)	(0.036)	(0.128)	(0.145)
Constant	0.829 ***	0.757 ***	0.542 ***	0.792 ***
	(0.01)	(0.008)	(0.036)	(0.029)
Observations	96	96	96	96
Adj-Rsq *p*-values (inclusion of health center fixed effects)	0.113 0.165	0.147 0.002 ***	0.219 0.003 ***	0.078 0.006 ***

Notes: Robust clustered standard errors in parentheses. All specifications control for health center fixed effects. Statistical significance: *** *p* < 0.01, * *p* < 0.1.

**Table 7:** Effects of signing CBHI contract on perceived quality in health care.

Variables	Problems of Drug Shortage	Problems of Budget Shortage	Waiting Time for Patient Card	Waiting Time for Seeing a Doctor/Nurse
CBHI*d2014	−0.278 **	−0.222 **	−12.86	0.95
	(0.106)	(0.108)	(9.67)	(7.51)
Time period—2014	0.083	0.083	13.56	−11.6 **
	(0.082)	(0.082)	(9.30)	(4.38)
Constant	0.146 ***	0.125 ***	12.45 ***	31.93 ***
	(0.027)	(0.029)	(1.55)	(2.35)
Observations	96	96	95	96
Adj-Rsq *p*-values (inclusion of health center fixed effects)	0.163 0.397	0.078 0.560	0.078 0.547	0.085 0.419

Notes: Robust clustered standard errors in parentheses. All specifications control for health center fixed effects. Statistical significance: *** *p* < 0.01, ** *p* < 0.05.

**Table 8:** Satisfaction with treatment received at contracted and non-contracted health centers.

	Health Care Service								
	2011			2012			2013		
	CBHI-Contracted	Non-Contracted	*p*-Value	CBHI-Contracted	Non-Contracted	*p*-Value	CBHI-Contracted	Non-Contracted	*p*-Value
Unsatisfied	0.07	0.01	0.02	0.11	0.09	0.64	0.073	0.03	0.15
Neutral	0.06	0.06	0.85	0.10	0.08	0.70	0.043	0.14	0.00
Satisfied	0.87	0.93	0.11	0.79	0.83	0.52	0.884	0.83	0.26
Observations	309	101		286	59		328	73	

Notes: *p*-values are for mean difference comparisons between contracted and non-contracted health centers for each year.

**Table 9:** Effect of CBHI contract on satisfaction with health care received.

Variables	1	2	3	4
CBHI-contracted health center*2012	0.035	0.031	0.047	0.061
	(0.065)	(0.066)	(0.071)	(0.072)
CBHI-contracted health center*2013	0.109 *	0.128 **	0.161 **	0.179 ***
	(0.057)	(0.058)	(0.063)	(0.063)
2012	−0.101 *	−0.101 *	−0.133 **	−0.127 **
	(0.056)	(0.057)	(0.061)	(0.062)
2013	−0.093 *	−0.105 **	−0.158 ***	−0.159 ***
	(0.051)	(0.052)	(0.056)	(0.055)
CBHI-contracted health center	−0.061 *	−0.025	−0.046	−0.015
	(0.033)	(0.035)	(0.039)	(0.040)
Constant	0.929 ***	1.033 ***	1.026 ***	1.076 ***
	(0.026)	(0.062)	(0.071)	(0.086)
Observations	1134	1124	961	960
Adj-Rsq	0.008	0.028	0.079	0.108

Notes: Additional controls in column 2 are socioeconomic status and demographic characteristics of the individuals; estimates in column 3 include the regressors in column 2 and add individual health status; estimates in column 4 include the regressors in column 3 and add participation in networks and participation in financial institutions and regional fixed effects. The set of variables included is described in Table A2. Detailed results are available on request. Robust standard errors in parentheses; statistical significance: *** *p* < 0.01, ** *p* < 0.05, * *p* < 0.1. The number of observations differs across columns 1 to 4 as data on the additional controls that are included are not always complete.

## Appendix A

**Table A1:** Description of variables: health center analysis.

Variable	Description
Variables	
Monthly volume of outpatients	Number of patients utilizing outpatient service in a month
Patient card revenue	Health facility’s annual revenue from fees paid for patient card
Diagnosis revenue	Health facility’s annual revenue from fees paid for diagnosis and tests
Drug revenue	Health facility’s annual revenue from sale of drugs
Government budget revenue	Health facility’s annual revenue from government budget allocation
Availability of essential drugs	Availability of eighteen essential drugs (ranges from 9 to 18)
Availability of medical facility/equipment	Availability of twenty-one types of medical equipment/facilities (ranges from 9 to 21)
Water supply	Health facility has adequate water supply (1 = yes)
Electricity access	Health facility has electricity access (1 = yes)
Waiting time to obtain medical card	Average waiting time (in minutes) before getting patient card (based on the response of exit interviews of five patients per facility)
Waiting time to see a medical professional	Average waiting time (in minutes) before seeing a medical professional, doctor/nurse (based on the response of exit interviews of five patients per facility)
Shortage/poor supply of drugs	The facility has shortage/poor supply of drugs problem (1 = yes it has the problem)
Shortage of budget	The facility has inadequate allocation of budget problem (1 = yes it has the problem)
Overall self-assessed quality of health care provided	Self-assessment of the respondent (typically the head of the facility) about the overall quality of health care services provided by the facility (1 = yes, the facility provides quality services)
CBHI	Health center is affiliated to CBHI scheme
Head’s education level	The maximum level of education the head of the facility has attended (1 = degree and above)
Head’s age	Age in years of the head of the health facility
Head’s training	Head has received training in health service management (1 = yes)
Head’s experience	Head has previously worked as head in another health facility (1 = yes)
Total number of staff	Number of medical and support staff in the health center

**Table A2:** Description of variables: household.

Variable	Description
**Outcome variable**	
Satisfaction with care	Satisfaction with the health care sought (1 = unsatisfied, 2 = neutral, 3 = satisfied)
**Explanatory Variables**	
CBHI	Health center is affiliated to CBHI scheme
*Socioeconomic status*	
Consumption quintiles	Classification of individuals based on monthly consumption expenditure (in Birr) excluding health care spending (poorest (1st) quintile), 2nd quintile, 3rd quintile, 4th quintile, richest (5th) quintile)
Education	Education level of an individual (no education, informal education, primary education, secondary and above education)
Land cultivated	Size of land cultivated in hectares
Household shock experience	Experience of any type of shock (health, natural, economic, social, institutional, market or other) in the twelve months preceding the survey.
*Demographic traits*	
Age	Age in complete years
Male	Male
Household size	Number of household members
Religion	Religion identifier (1 = Orthodox Christian, 2 = Protestant, 3 = Muslim, 4 = Other than 1, 2, 3)
**Health status**	
SAH-good	Self-assessed health is rated as good (includes very good and excellent)
SAH- not good	Self-assessed health is rated as not good (includes average)
Past illness event	Total number of days ill in the two months preceding the survey
Chronic Illness	Disease symptoms have persisted for more than 30 days
***Financial participation and networks***	
Savings in bank account	At least one member of the household has savings in bank account
Member of iqqub	At least one member of the household participates in iqqub
Member of credit and saving association	At least one member of the household participates in credit and saving association
Official position held	At least one member of the household held or still holds official (kebele or traditional) position.
Region	Region where the respondent is located

**Table A3:** Effect of CBHI contract on satisfaction with health care received: 2011 and 2013.

Variables	1	2	3	4
CBHI-contracted health center*2013	0.109 *	0.128 **	0.162 **	0.171 ***
	(0.057)	(0.058)	(0.062)	(0.063)
2013	−0.093 *	−0.107 **	−0.164 ***	−0.160 ***
	(0.051)	(0.051)	(0.055)	(0.055)
CBHI-contracted health center	−0.061 *	−0.066 *	−0.089 **	−0.064
	(0.033)	(0.035)	(0.039)	(0.041)
Constant	0.929 ***	1.045 ***	1.044 ***	1.079 ***
	(0.026)	(0.065)	(0.078)	(0.095)
Observations	803	802	677	676
Adj-Rsq	0.001	0.037	0.091	0.110

Notes: Additional controls in column 2 are socioeconomic status and demographic characteristics of the individuals; estimates in column 3 include the regressors in column 2 and add individual health status; estimates in column 4 include the regressors in column 3 and add participation in networks and participation in financial institutions and regional fixed effects. The set of variables included is described in Table A2. Robust standard errors in parentheses; statistical significance: *** *p* < 0.01, ** *p* < 0.05, * *p* < 0.1. The number of observations differs across columns 1 to 4 as data on the additional controls that are included are not always complete.
